# MicroRNAs miR-124 and miR-135a are potential regulators of the mineralocorticoid receptor gene (*NR3C2*) expression

**DOI:** 10.1016/j.bbrc.2009.11.128

**Published:** 2010-01-01

**Authors:** Siim Sõber, Maris Laan, Tarmo Annilo

**Affiliations:** Institute of Molecular and Cell Biology, University of Tartu, Riia 23, 51010 Tartu, Estonia

**Keywords:** MicroRNA, NR3C2, Mineralocortocoid receptor, Blood pressure

## Abstract

MicroRNAs (miRNAs) comprise a post-transcriptional layer of gene regulation shown to be involved in diverse physiological processes. We aimed to study whether regulatory networks that determine susceptibility to hypertension may involve a miRNA component. Screening of loci, involved in renal water–salt balance regulation, highlighted the mineralocorticoid receptor gene *NR3C2* as a potential target for several miRNAs. A luciferase assay demonstrated that miR-124 and miR-135a suppress *NR3C2* 3′UTR reporter construct activity 1.5- and 2.2-fold, respectively. As the tested miRNAs did not reduce the levels of target mRNA, we suggest that the binding of miR-124 and miR-135a to *NR3C2* 3′UTR contributes to the translational, not transcriptional regulation of the gene. Co-expression of two different miRNAs did not increase the repression of the reporter gene, indicating no additive or synergistic effects between the tested miRNAs.

Our results demonstrate that by repressing the mineralocorticoid receptor gene *NR3C2*, miR-124 and miR-135a could participate in the regulation of renin–angiotensin–aldosterone system and thereby might be involved in blood pressure regulation.

## Introduction

MicroRNAs (miRNAs), small noncoding RNA molecules, are endogenous post-transcriptional regulators that function as guide molecules, pairing with partially or fully complementary motifs in 3′UTRs of their target mRNAs [1]. In animal cells, the prevalent effect of miRNAs is translational repression of the target mRNA without affecting the levels of mRNA itself, although the mechanisms that induce target mRNA degradation by RNA induced silencing complex mediated cleavage [2] or rapid deadenylation [3] and decapping [4] have also been reported. Recently, transcriptional and post-transcriptional levels of gene regulation have been combined into the hypothesis that combinatorial control by transcription factors and miRNAs may be involved in regulation of the majority of cellular pathways [5,6]. Involvement of miRNAs in fundamental biological processes raises the hypothesis that alterations in miRNA-controlled pathways may cause developmental abnormalities and various human disorders. Indeed, it has been shown that miRNAs can play an important role in etiology of cancer and other diseases [7]. Despite that, the functional role of most of the miRNAs in human physiology is still poorly understood.

Essential hypertension is a complex disorder, caused by the interplay between many genetic variants, gene–gene interactions and environmental factors [8,9]. A number of genetic studies have been conducted to unscramble the complicated blood pressure-regulating genetic networks, resulting in a large number of candidate genes, sequence variants and quantitative trait loci, mostly with modest effect [8,10,11]. The most consistent results have been achieved in revealing the genetic component of familial hyper- or hypotension. So far, all genes found to cause Mendelian forms of blood pressure disturbances map to the physiological pathway regulating water–salt balance in kidneys [12].

Recent studies have reported that single-nucleotide polymorphisms within the miRNA target sites can modify miRNA binding, alter the normal level of target gene expression and contribute to the pathological phenotype [13,14]. For example, the 3′UTR of angiotensin II receptor I (*AGTR1*) contains the single-nucleotide polymorphism (SNP) rs5186 located within the target site for miR-155. The C allele at this position has been associated with several cardiovascular pathologies, including hypertension [15–17]. Two independent studies have shown that this allele drastically decreases binding of miR-155, thereby resulting in elevated *AGTR1* levels and increasing a risk for cardiovascular disease [14,18]. However, reliable target prediction and experimental validation are still the limiting steps in studying the impact of miRNA-mediated gene regulation on human health.

We set out to study whether regulatory networks responsible for susceptibility to hypertension may include miRNAs. Using *in silico* screening of blood pressure candidate genes for possible interactions with miRNAs we identified mineralocorticoid receptor *NR3C2* (located in 4q31.23), a gene involved in familial hypertension and renal salt-balance maintenance, as a potential candidate for miRNA-mediated regulation and verified experimentally that it is a target for miR-124 and miR-135a.

## Materials and methods

*In silico analysis of 160 blood pressure candidate genes.* A list of 160 candidate genes reported to be involved in blood pressure regulation and hypertension was assembled based on literature search in publicly available databases (Table S1) [9]. This list of 160 genes was analyzed for miRNA binding sites within 3′UTRs using Targetscan 4.2 [19] data [20]. The significance of overrepresentation of target sites for each miRNA family was estimated using binomial tests and Bonferroni correction was applied to account for multiple testing. 3′UTRs of a subset of genes (*n* = 35) involved in regulating water–salt balance in kidneys and associated with Mendelian hyper- and hypotension (Table S1), was screened for miRNA binding sites using three alternative target prediction algorithms: Targetscan [20], miRanda [21,22] and picTar [23,24]. Targetscan (conserved target sites and miRNA families in human, mouse, rat and dog) and picTar prediction data was downloaded from UCSC genome browser tracks and miRanda predictions were downloaded from www.microrna.org [21]. Structure of miRNA-3′UTR duplexes was predicted using RNAhybrid [25,26].

*Construction of NR3C2 3′UTR reporter.* The full-length NR3C2 3′UTR was amplified with primers NR3C2-UTR-F and NR3C2-UTR-R (Table S3) using Smart-Taq Hot polymerase (Naxo). The product was digested with BcuI (Fermentas) and FseI (New England Biolabs) and ligated into XbaI (Fermentas) and FseI digested pGL3-Control vector (Promega).

*Cloning of miRNA expression plasmids.* 450–500 bp genomic regions containing miRNA genes were cloned into pQM-NtagA vector for expression under the control of a CMV promoter. We amplified the genomic sequences containing the following miRNA genes with the primers given in Table S3: miR-124-1, miR-135a-2, miR-19b-1, miR-30e, and miR-130a. PCR products were digested with XbaI and BglII and ligated into the respective sites of the pQM-NtagA (Quattromed) vector to yield miRNA expression plasmids: pMir124, pMir135a, pMir19b, pMir30e and pMir130a.

*Determination of microRNA expression levels.* 2.8 × 10^5^ HeLa cells were plated on a 6-well cell culture plate in Opti-MEM (Invitrogen) medium with 10% FCS 24 h prior to transfection. Cells were transfected with 80 ng pRL-TK (Promega), 400 ng pGL3-NR3C2 and 1.6 μg pMir plasmid using Lipofectamine 2000 (Invitrogen) according to manufacturer’s instructions. Cells were harvested after 24 h and total RNA was extracted using mirVANA microRNA isolation kit (Ambion). miRNA expression was measured by qPCR using Taqman microRNA assays (Applied Biosystems) with ABI 7900HT Sequence Detection System (Applied Biosystems).

*Luciferase assays.* 8 × 10^4^ HeLa cells were plated into 24-well plates in Opti-MEM (Invitrogen) medium supplemented with 10% FCS 24 h prior to transfection. Cells were transfected with 20 ng pRL-TK (Promega), 100 ng pGL3-*NR3C2* and 400 ng of appropriate pMir plasmid using Lipofectamine 2000 (Invitrogen) according to manufacturer’s instructions. 200 ng + 200 ng of different pMir plasmids were used for miRNA co-expression assays. Cells were harvested after 24 h and analysed using Promega dual luciferase assay. Luciferase activity was measured using TD20/20 luminometer (Turner Designs). Assays were performed in three parallels, which were replicated three times. Statistical significance of results was calculated using two-tailed Mann–Whitney *U* test.

*Quantitative RT-PCR.* For RNA extractions 3 × 10^5^ cells were plated into 6-well plates in DMEM + 10% FCS without antibiotics. After 24 h cells were transfected with 80 ng pRL-TK, 400 ng pGL3-NR3C2 and 1.6 μg of pMir plasmid using Lipofectamine 2000. After 24 h incubation cells were harvested and RNA was extracted using Nucleospin RNA II kit (Macherey–Nagel). Three replicate transfections were performed.

cDNAs were synthesized from 200 ng of total RNA template using First Strand cDNA synthesis kit (Fermentas) and oligo dT primer according to manufacturer’s protocol. Quantitative PCR was performed in 96-well plates using SYBR Green ROX mix (ABGene) and ABI 7900HT Sequence Detection System (Applied Biosystems). The primers used for amplifications are listed in Table S3. Reactions were performed in 20 μl with 15 min initial denaturation at 95 °C. PCR was performed in three replicates. Data was analyzed using SDS 2.2.2 software (Applied Biosystems). Renilla luciferase was used as a reference for firefly luciferase reporter mRNA and endogenous *NR3C2* transcript data was normalized against *GAPDH*.

## Results

### Bioinformatic assessment of potential miRNA regulation of blood pressure candidate genes

First, we asked whether target sites for any miRNAs are overrepresented in 3′UTRs of genes controlling blood pressure compared to all human genes. We screened 3′UTRs of 160 blood pressure candidate genes (Table S1, [9]) using miRNA binding site prediction tool Targetscan 4.2 [20]. Binomial tests were performed for each miRNA family (groups of miRNAs with highly similar sequence). We found no evidence for miRNA binding sites that are specifically enriched in 3′UTRs of blood pressure-regulating genes (Table S2).

Next, we focused on 35 genes involved in renin–angiotensin–aldosterone system and other components of renal water–salt balance regulation (Table S1) and asked whether any of these genes could be a target for miRNA regulation. miRNA target identification quality was improved by parallel employment of three prediction programs: Targetscan [20], miRanda [22] and picTar [24]. This analysis identified miRNA targets in 13, 27 and 12 genes, respectively. Six genes (*ADD1*, *AGTR2*, *KCNJ1*, *NEDD4L*, *NR3C2*, and *SCNN1A*) contained target site predictions by all three algorithms (Table 1). All these genes have unusually long 3′UTRs (1026–2575 bp) (Table 1), a feature that has been associated with miRNA regulation [27]. Among these, the mineralocorticoid receptor gene, *NR3C2* contained the highest number of predicted miRNA target sites (23, 64, and 411 for Targetscan, PicTar and miRanda, respectively), indicating that *NR3C2* could be under especially intricate miRNA control.

### Selection of miRNAs for experimental validation

**Fig. 1:**
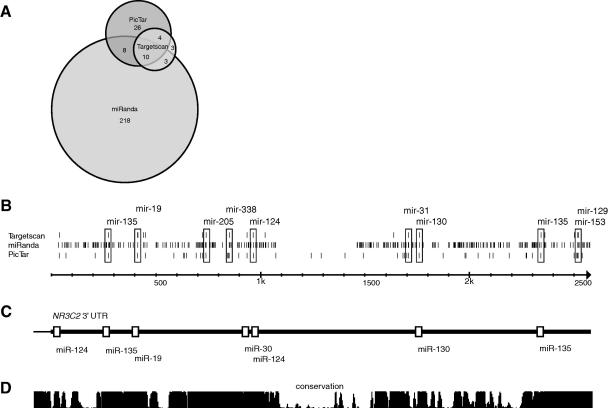
Graphic representation of predicted miRNA target sites in *NR3C2* 3′UTR. (A) Venn diagram showing the overlap of miRNA binding sites in *NR3C2* 3′UTR predicted by alternative algorithms (miRanda, Targetscan, PicTar). Numbers of non-overlapping predictions are indicated for each intersection. (B) Target site predictions from Targetscan, miRanda and Pictar datasets. Overlapping predictions are indicated with boxes. (C) Targetscan predictions of miRNA target sites selected for validation (depicted as white boxes). (D) Evolutionary conservation of DNA sequences in 17 vertebrate genomes from UCSC genome browser (http://genome.ucsc.edu/cgi-bin/hgGateway) phastCons track.

*In silico* prediction was followed by experimental validation of selected microRNA target sites. We focused on the mineralocorticoid receptor gene *NR3C2* as it has a highly conserved 3′UTR that is 2.5 kb in length and contains a remarkably high number of predicted miRNA target sites (Table 1 and Fig. 1).

The predicted target sites for miRNAs miR-124, miR-135, miR-30, miR-19 and miR-130 (Fig. 1C and Fig. S1) were considered to be best-supported based on the following criteria:

(1) Overlap between target prediction datasets: there was a considerable difference between the predictions from different datasets (Fig. 1A). However, 10 miRNA target sites in the 3′UTR of *NR3C2* were predicted by all three algorithms (Fig. 1B and C).
(2) Number of target site predictions in the 3′UTR of *NR3C2*: two putative target sites for miR-135 and three target sites for miR-124 were predicted by at least two algorithms.
(3) Quality of binding site predictions: we considered the scoring methods employed by Targetscan due to their solid statistical approach [28]. A target site for miR-30 was chosen for validation because it had the highest aggregate P_CT_ score (indicator of site conservation) for a single site in the Targetscan dataset.

miRNA variants with the best match to the target sequences (miR-124, miR-135a, miR-30e, miR-19b, and miR-130a) were selected for validation using a luciferase assay (Fig. S1).

### Exogenous and endogenous expression of selected miRNAs in HeLa cells

**Fig. 2:**
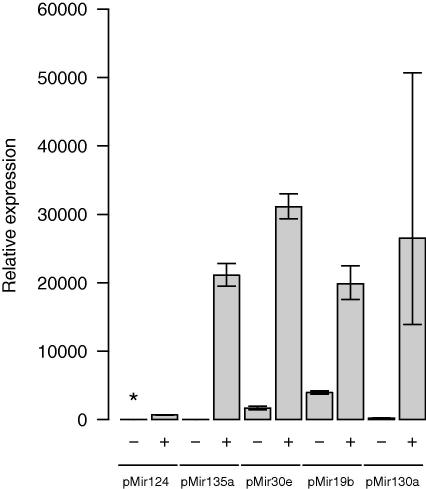
Expression of tested microRNAs in HeLa cells. Expression levels of endogenous and transfected microRNAs (miR-124, miR-135, miR-30e, miR-19b, miR-130a) were measured by qPCR (mean of 3–4 replicates) using ABI microRNA assays. Expression levels are normalized to endogenous miR-135, which was the miRNA with lowest detectable expression. Error bars represent 99.9% CI. ∗Expression was not detectable.

Expression plasmids for microRNAs miR-124, miR-135a, miR-30e, miR-19b, and miR-130a were constructed and tested for capability to produce microRNAs in HeLa cells. The expression of mature microRNAs was demonstrated by qPCR from all plasmids (Fig. 2). In HeLa cells, endogenous expression of microRNAs miR-30e, miR-19b, and miR-130a was detected, while endogenous miR-124 was undetectable and miR-135a was present in minuscule amounts (Fig. 2).

### miR-124 and miR-135a down-regulate *NR3C2* 3′UTR luciferase reporter

**Fig. 3:**
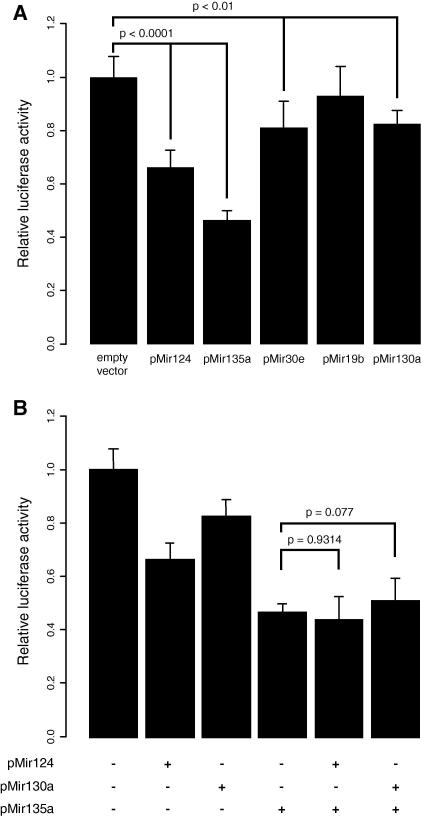
Luciferase reporter assays for NR3C2 3′UTR reporter construct in HeLa cells. (A) Reporter construct was cotransfected along with miRNA expression plasmid (miR-124, miR-135, miR-30e, miR-19b, miR-130a) and luciferase activity was assayed after 24 h after transfection using dual luciferase reporter assay. The results of nine replicate experiments are shown. Values were normalised to transfections with empty pQM-NtagA vector. *P* values were calculated using two-tailed Mann–Whitney *U* test. Error bars represent 95% CI. (B). Effects of miRNA co-expression on *NR3C2* 3′UTR reporter expression. *NR3C2* 3′UTR luciferase reporter plasmid was cotransfected with different combinations of expression constructs for miRNAs strongly repressing *NR3C2* reporter (miR-124 and miR-135a) and a miRNA exhibiting modest repression (miR-130a).

In order to validate the predicted miRNA binding sites, co-transfections of *NR3C2* 3′UTR luciferase reporter and expression vectors for the selected miRNAs (miR-124, miR-135a, miR-30e, miR-19b, and miR-130a) were performed. Transfections with miR-124 and miR-135a constructs resulted in significant repression of luciferase activity (*P* < 0.0001; Fig. 3A). The level of luciferase reporter signal was repressed 1.5-fold by miR-124 and 2.2-fold by miR-135a compared to transfections with an empty vector. miRNAs miR-30e and miR-130a repressed the activity of the luciferase reporter to a smaller extent (1.2-fold) (*P* < 0.01). miR-19b had no effect on the reporter activity. These results agree with bioinformatic predictions since the miRNAs with best-supported predictions (miR-124 and miR-135a) were found to cause the strongest repression.

### Co-transfection of multiple miRNAs does not enhance the repression of *NR3C2* reporter

Next, we tested whether simultaneous effect of more than one miRNA would enhance the silencing of *NR3C2.* It has been suggested that genes with a regulatory function are often subject to complex suppression by a number of interacting miRNAs [29]. We studied the combined effect of (i) two miRNAs with the strongest effect (miR-135a and miR-124), and (ii) miRNA with the strongest effect (miR-135a) and a miRNA with a modest effect on gene expression (miR-130a). In both experiments, we observed no additive or synergistic effects between different miRNAs (Fig. 3B). The co-expression of miR-135a and miR-124a suppressed the luciferase activity by the same degree (2.2-fold) as miR-135a alone.

### Tested miRNAs have no effect on mRNA transcript level

**Fig. 4:**
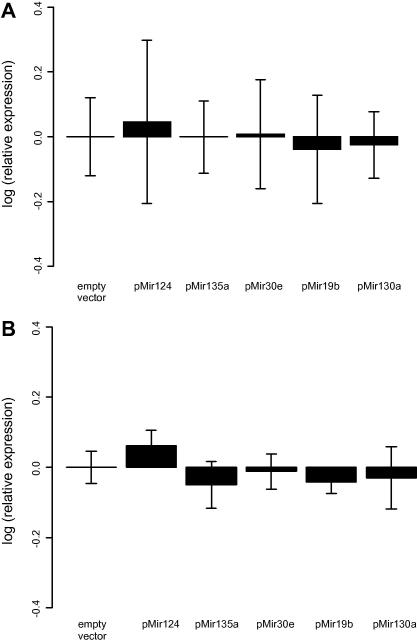
Quantification of the effects of different miRNAs on *NR3C2* transcript levels using qPCR with SYBR green. (A) Quantification of *NR3C2* 3′UTR reporter transcript levels. HeLa cells were cotransfected with *NR3C2* reporter and a miRNA expression plasmid (pMir124, pMir135, pMir30e, pMir19b, pMir130a). RNA was purified 24 h after transfection. Error bars represent 95% CI. (B) Quantification of endogenous *NR3C2* transcript. Error bars represent 99.9% CI.

We further asked whether the observed reduction of *NR3C2* luciferase reporter protein level results from the repression of mRNA expression. miRNAs are capable of down-regulating both mRNA transcription and translation levels of the target gene [1]. Therefore, we measured mRNA levels of both endogenous *NR3C2* and the *NR3C2* reporter construct using qPCR after transfection with miRNA expression constructs in HeLa cells. None of the transfected miRNAs were able to decrease the mRNA levels of either *NR3C2* reporter (Fig. 4A) or the endogenous *NR3C2* transcript (Fig. 4B) compared to cells transfected with an empty control vector. We note that even considering experimental uncertainty the variation in measured mRNA levels is not sufficient to account for the observed reduction in luciferase activity (Fig. 3A). Despite the presence of small amounts of *NR3C2* mRNA in HeLa cells, we were not able to test the effect of miRNA repression on the translation of endogenous NR3C2 since the level of endogenous protein in HeLa cell extracts was undetectable by Western blot assay (data not shown).

## Discussion

Based on several recent studies suggesting that miRNAs have a pivotal role in fine-tuning of regulatory circuits [30], we hypothesized that miRNA-mediated gene-expression modulation may contribute to the functioning of the genes that control blood pressure levels. To test this hypothesis, we screened hypertension candidate genes for putative miRNA target sites and experimentally verified the mineralocorticoid receptor *NR3C2* as a target for miR-124 and miR-135a. *NR3C2* is a ligand-dependent transcription factor that regulates the expression of ionic and water transporters in response to steroid hormones, primarily aldosterone. *NR3C2* can affect blood pressure directly by promoting salt retention in the kidney. In addition, *NR3C2* is also expressed in the brain (hypothalamus), where it mediates sympathetic regulation of blood volume homeostasis and influences salt appetite [31]. The critical role of *NR3C2* in salt balance regulation is shown in *NR3C2*-deficient mice that die in early neonatal period from severe sodium and water loss [32]. Also, mutation in *NR3C2* gene that alters the specificity of the receptor has been associated with autosomal dominant form of early-onset hypertension [33]. We found that the expression of *NR3C2* is reduced by miRNAs at the translational but not at the mRNA level. We suggest that by reducing the amount of mineralocorticoid receptor protein, miR-124 and miR-135a could attenuate signaling in the renin–angiotensin–aldosterone system and thus participate in regulation of blood pressure.

To investigate the possible role of miRNAs in pathogenesis of hypertension, the expression profile of miRNAs has been investigated in Dahl salt-sensitive rats [34]. It was shown that miRNA expression profile in the kidneys and heart of rats on normal and high-salt diet did not differ, indicating that salt loading does not have a significant effect on miRNA expression. In contrast, a large number of miRNAs are differentially expressed in cardiac hypertrophy [35,36], suggesting their important role in heart failure and contribution to cardiovascular disorders.

Among ∼1000 human miRNAs identified so far, miR-124 is one of the few with several experimentally verified targets. Expression profiling of mouse and human organs identified miR-124a as one of the brain-specific miRNAs [37] suggesting its role in neuronal differentiation. Since mineralocorticoid receptor is functionally important in the brain [34], miR-124 may have a direct role in regulation of *NR3C2* activity in central nervous system. Interestingly, Vreugdenhil and colleagues [38] have demonstrated that mir-124 can also regulate the glucocorticoid receptor (*NR3C1*), a paralog of NR3C2 by binding to a target site near the start of *NR3C1* 3′UTR. A homologous site is also present in the 3′UTR of *NR3C2* (Fig. 1C). miR-135 family is less studied but is known to be expressed in the kidney and at lower levels in the brain [39].

It has been demonstrated that the number of binding sites within a particular 3′UTR can determine the degree of repression [40]. Based on prediction algorithms, *NR3C2* contains at least three binding sites for miR-124 and two sites for miR-135 (Fig. 1). To test whether coordinate regulation occurs in case of *NR3C2*, we cotransfected different pairs of miRNAs along with *NR3C2* reporter construct. We did not observe neither additive nor synergistic effect in these experiments. It is possible that we did not observe combinatorial effects because of the high level of exogenous miRNAs in transfected cells that leads to the saturation of the repression pathway by a single miRNA. We note that the most significant repression in our experiments was generated by microRNAs with undetectable or low endogenous expression in HeLa cells (miR-124 and miR-135) (Fig. 2). We cannot exclude the scenario that the other tested miRNAs (miR-30e, miR-19b, and miR-130a) can also repress *NR3C2* but their effects were masked by the endogenous miRNAs. Variation in the extent of repression by different miRNAs may also be explained by several other factors, like the extent of binding site complementarity, sequence context near the site, location in respect to the stop codon and other miRNA target sites, as shown by Grimson and colleagues [28].

## Conclusions

In summary, bioinformatic screening showed no evidence of enrichment of specific miRNA target sites in the 3′UTRs of blood pressure controlling genes. Further inspection of individual genes identified *NR3C2* as a likely candidate for miRNA regulation. We showed that miRNAs miR-124 and miR-135a repress *NR3C2* translation *in vitro* without affecting its mRNA level. In addition we demonstrated that miR-124 and miR-135a do not act cooperatively in *NR3C2* reporter repression. These data imply that miRNAs can regulate mineralocorticoid receptor levels and thus may contribute to the modulation of the functioning of renin–angiotensin–aldosterone system.

## Figures and Tables

**Supplementary data:**
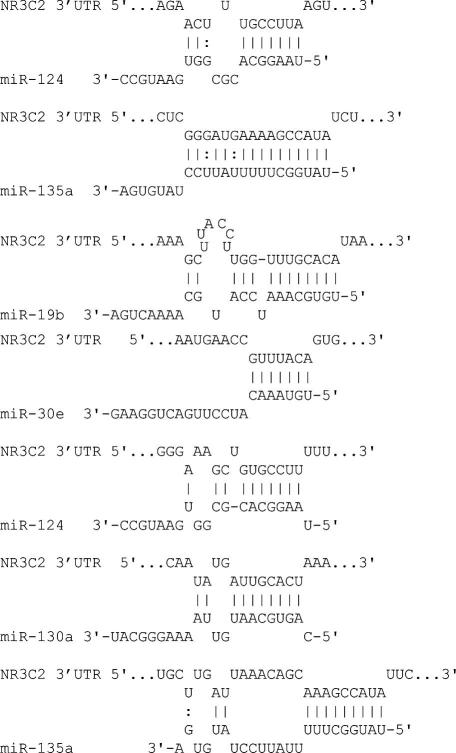
Sequences and predicted base-pairing of experimentally tested miRNAs with their predicted binding sites on *NR3C2* 3′UTR

**Table 1:** Number of predicted miRNA target sites in blood pressure candidate genes identified by Targetscan, PicTar and miRanda.

Gene	Targetscan	PicTar	MiRanda	3′UTR length (bp)
ADD1	4	5	34	1891
AGTR2	1	3	58	1195
KCNJ1	1	8	22	1105
NEDD4L	7	28	262	1888
NR3C2	23	64	411	2575
SCNN1A	2	2	131	1026
Average^a^	1.97	5.39	74.5	722

a Average target prediction counts and 3′UTR lengths are shown for 35 candidate genes (Supplementary Table S1).

## Appendix A Supplementary data

Supplementary data List of blood pressure candidate genes studied for microRNA binding sites

Supplementary data Top 10 miRNA families with largest discrepancy between expected and observed number of Targetscan miRNA target site predictions in 3′UTRs of 160 hypertension candidate genes.

Supplementary dat Primers used for PCR reactions

## References

[1] Bartel D.P. (2004). MicroRNAs: genomics, biogenesis, mechanism, and function. Cell.

[2] Yekta S., Shih I.H., Bartel D.P. (2004). MicroRNA-directed cleavage of HOXB8 mRNA. Science.

[3] Wu L., Fan J., Belasco J.G. (2006). MicroRNAs direct rapid deadenylation of mRNA. Proc. Natl. Acad. Sci. USA.

[4] Eulalio A., Rehwinkel J., Stricker M. (2007). Target-specific requirements for enhancers of decapping in miRNA-mediated gene silencing. Genes Dev..

[5] Chen K., Rajewsky N. (2007). The evolution of gene regulation by transcription factors and microRNAs. Nat. Rev. Genet..

[6] Zhou Y., Ferguson J., Chang J.T. (2007). Inter- and intra-combinatorial regulation by transcription factors and microRNAs. BMC Genomics.

[7] Blenkiron C., Miska E.A. (2007). miRNAs in cancer: approaches, aetiology, diagnostics and therapy. Hum. Mol. Genet..

[8] Cowley A.W. (2006). The genetic dissection of essential hypertension. Nat. Rev. Genet..

[9] Sober S., Org E., Kepp K. (2009). Targeting 160 candidate genes for blood pressure regulation with a genome-wide genotyping array. PLoS One.

[10] Org E., Eyheramendy S., Juhanson P. (2009). Genome-wide scan identifies CDH13 as a novel susceptibility locus contributing to blood pressure determination in two European populations. Hum. Mol. Genet..

[11] C. Newton-Cheh, T. Johnson, V. Gateva, et al., Genome-wide association study identifies eight loci associated with blood pressure, Nat. Genet. (2009). 10.1038/ng.361 PMC2891673 19430483

[12] Lifton R.P., Gharavi A.G., Geller D.S. (2001). Molecular mechanisms of human hypertension. Cell.

[13] Clop A., Marcq F., Takeda H. (2006). A mutation creating a potential illegitimate microRNA target site in the myostatin gene affects muscularity in sheep. Nat. Genet..

[14] Sethupathy P., Borel C., Gagnebin M. (2007). Human microRNA-155 on chromosome 21 differentially interacts with its polymorphic target in the AGTR1 3′ untranslated region: a mechanism for functional single-nucleotide polymorphisms related to phenotypes. Am. J. Hum. Genet..

[15] Cameron V.A., Mocatta T.J., Pilbrow A.P. (2006). Angiotensin type-1 receptor A1166C gene polymorphism correlates with oxidative stress levels in human heart failure. Hypertension.

[16] Kainulainen K., Perola M., Terwilliger J. (1999). Evidence for involvement of the type 1 angiotensin II receptor locus in essential hypertension. Hypertension.

[17] Osterop A.P., Kofflard M.J., Sandkuijl L.A. (1998). AT1 receptor A/C1166 polymorphism contributes to cardiac hypertrophy in subjects with hypertrophic cardiomyopathy. Hypertension.

[18] Martin M.M., Buckenberger J.A., Jiang J. (2007). The human angiotensin II type 1 receptor +1166 A/C polymorphism attenuates microrna-155 binding. J. Biol. Chem..

[19] TargetScan, www.targetscan.org.

[20] Lewis B.P., Burge C.B., Bartel D.P. (2005). Conserved seed pairing, often flanked by adenosines, indicates that thousands of human genes are microRNA targets. Cell.

[21] www.microrna.org.

[22] John B., Enright A.J., Aravin A. (2004). Human microRNA targets. PLoS Biol..

[23] PicTar, http://pictar.mdc-berlin.de/.

[24] Krek A., Grun D., Poy M.N. (2005). Combinatorial microRNA target predictions. Nat. Genet..

[25] RNAhybrid, http://bibiserv.techfak.uni-bielefeld.de/rnahybrid.

[26] Rehmsmeier M., Steffen P., Hochsmann M. (2004). Fast and effective prediction of microRNA/target duplexes. RNA.

[27] Stark A., Brennecke J., Bushati N. (2005). Animal microRNAs confer robustness to gene expression and have a significant impact on 3′UTR evolution. Cell.

[28] Grimson A., Farh K.K., Johnston W.K. (2007). MicroRNA targeting specificity in mammals: determinants beyond seed pairing. Mol. Cell.

[29] Shalgi R., Lieber D., Oren M. (2007). Global and local architecture of the mammalian microRNA-transcription factor regulatory network. PLoS Comput. Biol..

[30] Kim J., Inoue K., Ishii J. (2007). A MicroRNA feedback circuit in midbrain dopamine neurons. Science.

[31] de Kloet E.R., Van Acker S.A., Sibug R.M. (2000). Brain mineralocorticoid receptors and centrally regulated functions. Kidney Int..

[32] Berger S., Bleich M., Schmid W. (1998). Mineralocorticoid receptor knockout mice: pathophysiology of Na+ metabolism. Proc. Natl. Acad. Sci. USA.

[33] Geller D.S., Farhi A., Pinkerton N. (2000). Activating mineralocorticoid receptor mutation in hypertension exacerbated by pregnancy. Science.

[34] Naraba H., Iwai N. (2005). Assessment of the microRNA system in salt-sensitive hypertension. Hypertens. Res..

[35] Cheng Y., Ji R., Yue J. (2007). MicroRNAs are aberrantly expressed in hypertrophic heart: do they play a role in cardiac hypertrophy?. Am. J. Pathol..

[36] van Rooij E., Sutherland L.B., Liu N. (2006). A signature pattern of stress-responsive microRNAs that can evoke cardiac hypertrophy and heart failure. Proc. Natl. Acad. Sci. USA.

[37] Sempere L.F., Freemantle S., Pitha-Rowe I. (2004). Expression profiling of mammalian microRNAs uncovers a subset of brain-expressed microRNAs with possible roles in murine and human neuronal differentiation. Genome Biol..

[38] Vreugdenhil E., Verissimo C.S., Mariman R. (2009). MicroRNA 18 and 124a down-regulate the glucocorticoid receptor: implications for glucocorticoid responsiveness in the brain. Endocrinology.

[39] Hsu S.D., Chu C.H., Tsou A.P. (2008). Nucleic Acids Res..

[40] Doench J.G., Petersen C.P., Sharp P.A. (2003). siRNAs can function as miRNAs. Genes Dev..

