# Supplementary material for: MicroRNAs miR-124 and miR-135a are potential regulators of the mineralocorticoid receptor gene (NR3C2) expression
Source: Biochem Biophys Res Commun. 2010 Jan 1;391(1):727–32. doi: 10.1016/j.bbrc.2009.11.128 (PMC2806518; doi:10.1016/j.bbrc.2009.11.128)
Supplement: Supplementary data — List of blood pressure candidate genes studied for microRNA binding sites [file mmc1.pdf]

List of blood pressure candidate genes studied for microRNA binding sites - coordinates and 3'UTR lengths from UCSC genome browser (hg18 assembly)

Gene	Name	Chr	Start	End	3' UTR length (bp)
**Genes involved in renal water-salt balance**					
ACE	angiotensin I converting enzyme	17	58908166	58928711	252
ACSM3	SA hypertension-associated homolog	16	20682813	20715979	196
ADD1	adducin 1 (alpha)	4	2882590	2968794	1887
AGT	angiotensinogen preproprotein	1	227145006	227156676	602
AGTR1	angiotensin II receptor, type 1	3	149898356	149943489	886
AGTR2	angiotensin II receptor, type 2	X	115113885	115117701	1195
ATP1A1	Na+/K+ -ATPase alpha 1 subunit isoform b	1	116627878	116659436	652
BSND	barttin	1	55176638	55186485	163
CLCNKA	chloride channel Ka	1	16093792	16105850	391
CLCNKB	chloride channel Kb	1	16115658	16128782	391
CYP11B1	cytochrome P450, family 11, subfamily B	8	143950777	143958238	2014
CYP11B2	cytochrome P450, subfamily XIB polypeptide 2	8	143988977	143996261	1421
CYP17A1	cytochrome P450, family 17	10	104580280	104587280	165
HSD11B1	11-beta-hydroxysteroid dehydrogenase 1	1	206247945	206296689	546
HSD11B2	hydroxysteroid (11-beta) dehydrogenase 2	16	66022537	66028953	1151
KCNJ1	potassium inwardly-rectifying channel J1	11	128213125	128242478	1105
KLK1	kallikrein 1 preproprotein	19	56014216	56018855	435
NEDD4L	neural precursor cell expressed, developmentally	18	53862778	54216369	1888
NPPA	natriuretic peptide precursor A	1	11840042	11842101	290
NPPB	natriuretic peptide precursor B preproprotein	1	11851788	11853258	188
NPPC	natriuretic peptide precursor C	2	232615640	232616464	0
NPR1	natriuretic peptide receptor 1	1	150464186	150479536	573
NPR2	natriuretic peptide receptor B precursor	9	35782406	35799727	267
NR3C2	nuclear receptor subfamily 3, group C, member 2	4	149357525	149721128	2575
REN	renin precursor	1	200855605	200867122	196
SCNN1A	sodium channel, nonvoltage-gated 1 alpha	12	6326276	6354976	1024
SCNN1B	sodium channel, nonvoltage-gated 1, beta	16	23221141	23300117	494
SCNN1G	sodium channel, nonvoltage-gated 1, gamma	16	23101541	23135701	1410
SGK1	serum/glucocorticoid regulated kinase 1	6	134532082	134537695	1017
SLC12A3	solute carrier family 12, member 3	16	55456643	55504850	32
SLC14A2	solute carrier family 14 (urea transporter)	18	41448764	41517056	574
SLC22A2	solute carrier family 22 member 2	6	160608203	160650370	673
SLC8A1	solute carrier family 8	2	40250937	40569095	248
WNK1	WNK lysine deficient protein kinase 1	12	732993	888219	0
WNK4	WNK lysine deficient protein kinase 4	17	38186222	38202605	373
**Other blood pressure candidate genes**					
ABCC8	ATP-binding cassette, sub-family C, member 8	11	17371008	17455025	
ADD2	adducin 2	2	7090698	70800920	
ADD3	adducin 3 (gamma)	10	111757701	111885310	
ADM/AM	adrenomedullin	11	10283218	10285499	
ADORA1	adenosine A1 receptor	1	199828493	199868190	
ADORA2A	adenosine A2a receptor	22	23153646	23162878	
ADRA1A	alpha-1A-adrenergic receptor	8	26661584	26778839	
ADRA2A	alpha-2A-adrenergic receptor	10	112826911	112830560	
ADRB2	adrenergic, beta-2-, receptor, surface	5	148186349	148188381	
ADRB3	adrenergic receptor, beta 3	8	37939673	37943341	
AKR1B1	aldo-keto reductase family 1, member B1	7	133584362	133601143	
APOA1	apolipoprotein A-I preproprotein	11	116211679	116213548	
APOA2	apolipoprotein A-II preproprotein	1	158005156	158006491	
APOC2	apolipoprotein C-II precursor	19	50141083	50144657	

APOC3	apolipoprotein C-III precursor	11	116205834	116208997
APOC4	apolipoprotein C-IV	19	50137335	50140591
APOE	apolipoprotein E precursor	19	50100879	50104489
AQP2	aquaporin 2	12	48630796	48638929
AVP	arginine vasopressin-neurophysin II	20	3011203	3013370
AVPR1A	arginine vasopressin receptor 1A	12	61826483	61832857
AVPR1B	arginine vasopressin receptor 1B	1	202767011	202774698
AVPR2	arginine vasopressin receptor 2	X	152691217	152693486
BDKRB2	bradykinin receptor B2	14	95740950	95780536
BRS3	bombesin-like receptor 3	X	135295645	135300118
CACNA1C	calcium channel, voltage-dependent, L type,	12	2032725	2677376
CALCA	calcitonin isoform CALCA preproprotein	11	14944792	14950408
CMA1	chymase 1, mast cell preproprotein	14	24044552	24047311
CYP4A11	cytochrome P450, family 4, subfamily A	1	47106868	47119437
DBH	dopamine beta-hydroxylase precursor	9	133531039	133554020
DRD1	dopamine receptor D1	5	174800281	174803769
DRD2	dopamine receptor D2 isoform short	11	112785528	112851091
ECE1	endothelin converting enzyme 1	1	21291658	21362213
EDN1	endothelin 1	6	12398645	12404761
EDN2	endothelin 2	1	41613542	41619390
EDN3	endothelin 3	20	57308894	57334441
EDNRA	endothelin receptor type A	4	148759735	148823710
EDNRB	endothelin receptor type B	13	77367617	77447665
F2R	coagulation factor II receptor precursor	5	76047547	76067054
GAL	galanin preproprotein	11	68208559	68215218
GALR	galanin receptor 1	18	73090721	73111081
GCG	glucagon preproprotein	2	162824896	162834264
GCK	glucokinase	7	43957110	44002278
GFPT1	glucosamine-fructose-6-phosphate	2	69464058	69525976
GH1	growth hormone 1	17	59348296	59349930
GH2	growth hormone 2	17	59311306	59312955
GIPR	gastric inhibitory polypeptide receptor	19	50863342	50877557
GLP1R	glucagon-like peptide 1 receptor	6	39124595	39163497
GNAI1	guanine nucleotide binding protein (G protein)	7	79408791	79493372
GNB3	guanine nucleotide-binding protein, beta-3	12	6819636	6826817
GYS1	glycogen synthase 1 (muscle)	19	54163195	54188361
GYS2	glycogen synthase 2 (liver)	12	21580390	21649048
HP	haptoglobin	16	70646009	70652458
IAPP	islet amyloid polypeptide precursor	12	21417085	21423683
ICAM1	intercellular adhesion molecule 1 precursor	19	10242779	10258291
ICAM2	intercellular adhesion molecule 2 precursor	17	59433688	59437839
ICAM3	intercellular adhesion molecule 3 precursor	19	10305454	10311300
IGF1	insulin-like growth factor 1	12	101292143	101376791
IGF2	insulin-like growth factor 2	11	2106924	2127409
INS	proinsulin precursor	11	2137585	2139015
INSR	insulin receptor	19	7063266	7245011
KCNJ11	potassium inwardly-rectifying channel J11	11	17363374	17366782
KCNJ6	potassium inwardly-rectifying channel J6	21	37918657	38210566
KCNK2	potassium channel, subfamily K, member 2	1	211567280	211798831
KNG	kininogen 1	3	187917822	187944443
LDLR	low density lipoprotein receptor precursor	19	11061132	11105490
LEP	leptin precursor	7	127475282	127491632
LEPR	leptin receptor	1	65598339	65814841
LPL	lipoprotein lipase precursor	8	19841058	19869049

LRP8	low density lipoprotein receptor-related protein	1	53423233	53505842
MTHFR	5,10-methylenetetrahydrofolate reductase	1	11780053	11800381
NOS1	nitric oxide synthase 1 (neuronal)	12	116113699	116262302
NOS2A	nitric oxide synthase 2A	17	23107920	23151682
NOS3	nitric oxide synthase 3 (endothelial cell)	7	150125795	150149323
NPR3	natriuretic peptide receptor C/guanylate cyclase	5	32747422	32823009
NPY	neuropeptide Y	7	24097049	24104717
NPY1R	neuropeptide Y receptor Y1	4	164602722	164611353
NR3C1	nuclear receptor subfamily 3, group C, member 1	5	142637689	142795270
PDX1	pancreatic and duodenal homeobox 1	13	27392157	27397394
PLA2G1B	phospholipase A2, group IB	12	119222635	119228312
PNMT	phenylethanolamine N-methyltransferase	17	35078033	35080254
PPP1CA	protein phosphatase 1, catalytic subunit, alpha	11	66922228	66925952
PPP1CC	protein phosphatase 1, catalytic subunit, gamma	12	109620464	109643387
PRCP	prolylcarboxypeptidase	11	82213058	82289205
PRKCE	protein kinase C, epsilon	2	45790694	46326779
PRKCQ	protein kinase C, theta	10	6509111	6662244
PTGER2	prostaglandin E receptor 2 (subtype EP2), 53kDa	14	51850863	51865070
PTGER3	prostaglandin E receptor 3, subtype EP3	1	71030057	71225512
PTGIR	prostaglandin I2 (prostacyclin) receptor (IP)	19	51815566	51820194
PTGIS	prostaglandin I2 (prostacyclin) synthase	20	47553818	47618114
PTGS1	prostaglandin-endoperoxide synthase 1	9	122212783	122237535
PTGS2	prostaglandin-endoperoxide synthase 2 precursor	1	183372627	183381213
PTHLH	parathyroid hormone-like hormone	12	28002285	28016183
PTHR1	parathyroid hormone receptor 1 precursor	3	46894240	46920290
RENBP	renin binding protein	X	152721570	152731079
SCN5A	voltage-gated sodium channel type V alpha	3	38564558	38666167
SCNN1D	sodium channel, nonvoltage-gated 1, delta	1	1257499	1267332
SELE	selectin E precursor	1	166423440	166434837
SERPINA4	serine (or cysteine) proteinase inhibitor, clade	14	94097536	94105994
SLC12A1	sodium potassium chloride cotransporter 2	15	46287190	46382416
SLC12A4	solute carrier family 12, member 4	16	66535731	66560026
SLC2A1	solute carrier family 2	1	43060612	43093594
SLC2A2	solute carrier family 2	3	172196839	172227470
SLC2A3	solute carrier family 2	12	7963094	7980138
SLC2A4	glucose transporter 4	17	7125778	7132090
SLC4A1	solute carrier family 4, anion exchanger	17	39682566	39700993
SLC4A2	solute carrier family 4, anion exchanger, member	7	150194387	150211257
SLC4A3	solute carrier family 4, anion exchanger	2	220317792	220332197
SLC4A4	solute carrier family 4, sodium bicarbonate	4	72569852	72802834
SLC5A2	solute carrier family 5, member 2	16	31401940	31409590
SLC6A2	solute carrier family 6 member 2	16	54248057	54295199
SLC8A2	solute carrier family 8 member 2	19	52623735	52666934
SLC9A1	solute carrier family 9, isoform A1	1	27109449	27165543
SLC9A2	solute carrier family 9, member 2	2	102694684	102786325
SLC9A5	solute carrier family 9	16	65840356	65863594
TBXA2R	thromboxane A2 receptor	19	3545504	3557658
TBXAS1	thromboxane A synthase 1	7	138982136	139173186
TGFB1	transforming growth factor, beta 1	19	46528491	46551656
TH	tyrosine hydroxylase	11	2141736	2149611
TRH	thyrotropin-releasing hormone	3	131176261	131179478
TRHR	thyrotropin-releasing hormone receptor	8	110168902	110200989
TRIP10	thyroid hormone receptor interactor 10	19	6690707	6702528
UCP3	uncoupling protein 3	11	73388985	73397778

VEGFA	vascular endothelial growth factor A	6	43845931	43862199
VEGFB	vascular endothelial growth factor B	11	63758842	63762834
VEGFC	vascular endothelial growth factor C	4	177979840	178089044
