# Supplementary material for: MicroRNAs miR-124 and miR-135a are potential regulators of the mineralocorticoid receptor gene (NR3C2) expression
Source: Biochem Biophys Res Commun. 2010 Jan 1;391(1):727–32. doi: 10.1016/j.bbrc.2009.11.128 (PMC2806518; doi:10.1016/j.bbrc.2009.11.128)
Supplement: Supplementary data — Top 10 miRNA families with largest discrepancy between expected and observed number of Targetscan miRNA target site predictions in 3′UTRs of 160 hypertension candidate genes. [file mmc2.pdf]

Top 10 miRNA families with largest discrepancy between expected and observed number of Targetscan miRNA target site predictions in 3'UTRs of 160 hypertension candidate genes.

P values were obtained with binomial tests. None of the tests reached Bonferroni corrected significance level ($\alpha = 0.05 / 162 = 3.09x10^{-4}$).

	No of miRNA target site predictions			
	Observed			
miRNA family	All genes	Candidate genes	Expected	P value
miR-140	180	6	1.555	0.0053
miR-122	144	4	1.244	0.0377
miR-365	168	4	1.451	0.0597
miR-214	402	7	3.473	0.0632
miR-19	770	11	6.652	0.0756
miR-150	183	4	1.581	0.0762
miR-200bc/429	684	10	5.909	0.0778
miR-455	126	3	1.088	0.0973
miR-185	203	4	1.754	0.1014
miR-496	723	10	246	0.1019
