# Supplementary material for: MicroRNAs miR-124 and miR-135a are potential regulators of the mineralocorticoid receptor gene (NR3C2) expression
Source: Biochem Biophys Res Commun. 2010 Jan 1;391(1):727–32. doi: 10.1016/j.bbrc.2009.11.128 (PMC2806518; doi:10.1016/j.bbrc.2009.11.128)
Supplement: Supplementary dat — Primers used for PCR reactions [file mmc3.pdf]

Oligonucleotide primers used for PCR reactions.

Primer	Sequence	Underlined restriction site
**Construction of *NR3C2* 3’UTR reporter**		
NR3C2-UTR-F	5’-GAC TAG TCC AAG CCG CTC TAC TTC CAC-3’	BcuI
NR3C2-UTR-R	5’-GAC TGA GGC CGG CCT TGC TCC AAA ACA ACA GGT G-3’	FseI
**Cloning of miRNA expression plasmids**		
miR-124-1-F	5’-GAT CTA GAC TGT CTG TCA CAG GCT GCA C-3’	XbaI
miR-124-1-R	5’-GAA GAT CTG ATG CTG TGG TCC CTT CCT C-3’	BglII
miR-135a-2-F	5’-GAT CTA GAT GGC TGA ATG TTG CTG AGT G-3’	XbaI
miR-135a-2-R	5’-GAA GAT CTG AAC ACC AGG CAG GTA GCA G-3’	BglII
miR-19b-1-F	5’-GAT CTA GAT GAT GGT GGC CTG CTA TTT C-3’	XbaI
miR-19b-1-R	5’-GAA GAT CTT CAC AAT CCC CAC CAA ACT C-3’	BglII
miR-30e-F	5’-GAT CTA GAA GGG CCT TTG GAT TAG CAA G-3’	XbaI
miR-30e-R	5’-GAA GAT CTC AGC CCA CAG AAA ACA AGG A-3’	BglII
miR-130a-F	5’-GAT CTA GAG TCA GGG GGT TGG TGA AGA-3’	XbaI
miR-130a-R	5’-GAA GAT CTG GGC TGT TAC CTC CCA GAA G-3’	BglII
**Quantitative RT-PCR**		
firefly luciferase-F	5’-TGC ACA TAT CGA GGT GGA CAT C-3’	
firefly luciferase-R	5’-TGC CAA CCG AAC GGA CAT-3’	
Renilla luciferase-F	5’-GCT TAT CTA CGT GCA AGT GAT GAT TT-3’	
Renilla luciferase-R	5’-GAA ACT TCT TGG CAC CTT CAA CA-3’	
NR3C2-F	5’-AAA CAT ACG AAC AGC CAA TTT CTC T-3’	
NR3C2-R	5’-AGA CTG ATG CAT CTT CTC TTC ATT AAA G-3’	
GAPDH-F	5’-GCA CCG TCA AGG CTG AGA AC-3	
GAPDH-R	5’-GGA TCT CGC TCC TGG AAG ATG-3’	
